# Evaluation of Hepatitis B core-related antigen (HBcrAg) as a biomarker in cohorts from the United Kingdom and South Africa

**DOI:** 10.1016/j.jinf.2025.106601

**Published:** 2025-08-21

**Authors:** Louise O. Downs, Marion Delphin, Marije van Schalkwyk, Susan Hugo, Sheila F. Lumley, Elizabeth Waddilove, Tingyan Wang, Jacqueline Martin, Catherine de Lara, Arran Babbs, Monique I. Andersson, Richard H. Glashoff, M. Azim Ansari, Kosh Agarwal, Geoffrey Dusheiko, Jantjie Taljaard, Wolfgang Preiser, Eleanor Barnes, Gavin Kelly, Ivana Carey, Yusuke Shimakawa, Tongai G. Maponga, Philippa C. Matthews

**Affiliations:** a Nuffield Department of Medicine, University of Oxford, UK; b Department of Infectious Diseases and Microbiology, Oxford University Hospitals NHS Foundation Trust, Oxford, UK; c The Francis Crick Institute, 1 Midland Road, London, UK; d Division of Infectious Diseases, Department of Medicine, Stellenbosch University and Tygerberg Hospital, Cape Town, South Africa; e Department of Hepatology, Oxford University Hospitals NHS Foundation Trust, Oxford, UK; f Division of Medical Microbiology & Immunology, Stellenbosch University and National Health Laboratory Service Tygerberg Business Unit, Cape Town, South Africa; g Radcliffe Department of Medicine, University of Oxford, Oxford, UK; h Department of Hepatology and Oxford NIHR Biomedical Research Centre, Oxford University Hospitals NHS Foundation Trust, Oxford, UK; i Institute of Liver Studies, King’s College Hospital, London, UK; j Division of Medical Virology, Stellenbosch University and National Health Laboratory Service Tygerberg Business Unit, Cape Town, South Africa; k Institut Pasteur, Université Paris Cité, Unité d′Épidémiologie des Maladies Émergentes, Paris, France; l Department of Infectious Diseases, University College London Hospitals, Euston Road, London, UK; m Division of Infection and Immunity, University College London, Gower Street, London, UK

**Keywords:** HBV, Hepatitis B, Monitoring, Biomarker, HbcrAg, Core related antigen

## Abstract

**Objectives:**

We set out to evaluate Hepatitis B core-related antigen (HBcrAg) as a proxy for hepatitis B (HBV) viral load (VL) and liver disease in two different population settings.

**Methods:**

We undertook a cross-sectional retrospective observational study using samples and data from adults living with chronic HBV infection from the United Kingdom (UK, n=142) and South Africa (SA, n=211). We assessed HBcrAg distribution, relationship with other biomarkers, and risk stratification performance, applying point of care test (POCT) thresholds.

**Results:**

SA and UK cohorts differed by ethnicity, HIV coinfection, HBeAg-positivity and proportion with HBV VL > 200,000 IU/ml (all p < 0.001). HBcrAg positively correlated with alanine aminotransferase (ALT) (in both settings p < 0.01), and fibrosis/cirrhosis by APRI score (p=0.03 in UK, p=0.008 in SA), but not with elastography or FIB-4 scores. HBcrAg ≥4.3 log10U/ml (POCT threshold) was 100% sensitive and 92% specific for predicting VL > 200,000 IU/ml in the UK cohort, compared to 94% sensitive and 86% specific in the SA population.

**Conclusions:**

HBcrAg correlated with VL, but less so with liver disease. Use of this biomarker needs tailoring for use in diverse populations.

## Introduction

A WHO report published in 2024 estimated 254 million people worldwide are living with chronic hepatitis B infection (CHB), resulting in ~1.1 million deaths per year as a result of liver disease, including cirrhosis and hepatocellular carcinoma (HCC).^1^ International targets have been set for elimination of viral hepatitis as a global public health threat by 2030,^2^ but there is a shortage of reliable, accessible and affordable clinical tools to predict outcomes of CHB and its treatment, so new approaches are needed. More precise identification of people at risk of liver complications could inform individualised care, such as enhanced surveillance, treatment or preventive interventions, while also providing evidence to underpin resource allocation.

HBV treatment guidelines stratify individuals for antiviral therapy with nucleos/tide analogue (NA) drugs based on a combination of serum markers and liver imaging.^3,4^ Hepatitis B ‘e′ antigen (HBeAg) positivity, high HBV DNA viral load (VL) and high hepatitis B surface antigen (HBsAg) titres are all associated with progression to cirrhosis and HCC.^5–7^ WHO treatment guidelines released in 2024 relaxed criteria for treatment,^8^ such that a wider population became treatment eligible, based on assessment with a less complex algorithm.

The performance of clinical laboratory liver markers, such as alanine aminotransferase (ALT), for predicting liver fibrosis is inconsistent; in some studies, ALT has a high sensitivity for predicting liver fibrosis,^9^ whilst in others it was a very poor predictor.^10^ Furthermore, laboratory markers are not universally available due to infrastructure and resource constraints. There is a need for cheaper and more widely accessible tools for monitoring and predictors of outcomes (both on and off NA treatment). Meanwhile, as the search for cure therapies continues, there is also a need to develop biomarkers that can support a mechanistic understanding of liver pathology and approaches to monitoring,^11^ in parallel with an increasing focus on biomarkers that might help support safe treatment cessation in selected individuals.^12^

Hepatitis B core-related antigen (HBcrAg) is a circulating serum biomarker that combines HBeAg, hepatitis B core antigen (HBcAg), and a small 22kd protein called p22cr.^13,14^ HBcrAg is a surrogate marker of active viral transcriptional activity, shown through comparison of serum HBcrAg levels and covalently closed circular DNA (cccDNA) quantification in liver biopsies,^11,13,15^ and also correlates with peripheral HBV VL and HBeAg status.^16,17^ Despite its overlap with HBeAg, in a Chinese study, over 50% of individuals testing HBeAg-negative (primarily with undetectable VL) still had detectable HBcrAg after 7 years of follow-up.^18^ HBcrAg has been associated with non-invasive scores of liver inflammation or fibrosis (Aspartate aminotransferase (AST) to Platelets Ratio Index (APRI) and Fibrosis-4 (FIB-4) score), ALT levels,^19,20^ hepatic necroinflammation,^15,16^ HCC,^21^ liver flares after treatment cessation^22^ and ‘disease stages’.^23^ In a Japanese cohort, followed up for over 10 years, HBcrAg was a better predictor of HCC than HBV DNA.^24^

HBcrAg could therefore be of utility as an additional biomarker to add to the existing tool-kit and may have a particular place in risk-stratification when HBV VL is low or when tools for VL quantification are not available. For these reasons, WHO guidelines highlight the potential use of HBcrAg assays,^8^ while recognising that there are no commercially available assays so activity remains confined to the research domain at present. There are currently few evaluations of HBcrAg representing diverse populations. Most existing studies are in Asia or Europe, thus neglecting the representation of individuals of African descent (and also overlooking the HBV genotypes that circulate most frequently in African populations).^25,26^ One study in Africa assessed quantitative HBcrAg, (in the PROLIFICA cohort, based in the Gambia), finding that HBcrAg could predict clinically important HBV VL thresholds.^27^

To move towards more accessible HBcrAg assessment, a point-of-care test (POCT) has been developed and validated^28^ to identify highly viremic individuals, with an estimated threshold for positivity at 4.3 log IU/ml. This was assessed in the PROLIFICA cohort and in one other cohort from Kenya.^29^ This POCT could enhance timely clinical assessment, support early treatment decisions, and promote linkage to care and continuity of follow-up.^28,30^

**Fig. 1:**
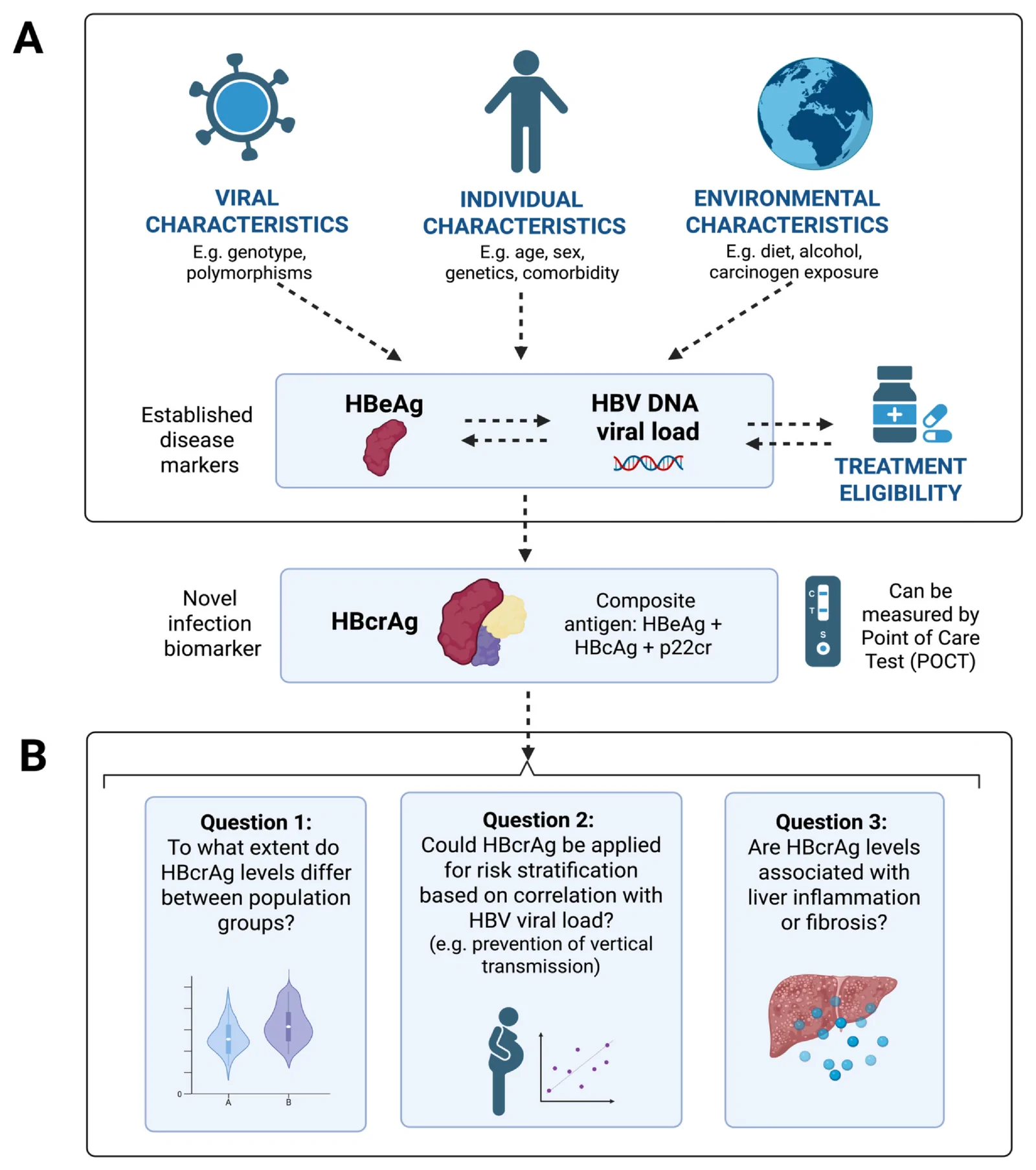
Schematic to show the known and potential relationships between HBcrAg and other viral, host and environmental factors. (A) External factors which may influence HBcrAg; (B) Research questions to determine the potential utility of HBcrAg measurement. Created in BioRender with a licence to publish. Matthews, P. (2025). https://BioRender.com/szcssic.

To add to the body of available evidence for the use of HBcrAg as a biomarker, we studied two cohorts recruited in the United Kingdom (UK) and South Africa (SA) (Fig. 1). We set out to quantify HBcrAg, and to describe its relationship with host characteristics and routine baseline clinical biomarkers (HBeAg and VL) within and between cohorts. We further explored how HBcrAg might have utility in predicting: (a) laboratory markers of liver inflammation (ALT); (b) clinical liver disease progression, including fibrosis and cirrhosis; and (c) clinically relevant VL thresholds. On this basis, we aim to contribute to the dialogue about the real-world use of HBcrAg to support delivery of interventions for HBV surveillance, treatment and prevention.

## Methods

### Study recruitment and collection of samples and data

We undertook a cross-sectional, retrospective observational study, using serum samples obtained from adults with CHB enrolled at Oxford University Hospitals (OUH) NHS Foundation Trust (UK, n=142), and from the ‘OXSA-Hep’ Cohort in Cape Town and Bloemfontein (SA; n=211).^31^ Study settings and recruitment have been previously described for the UK^32,33^ and SA.^31,34,35^ Ethics approval was provided by Oxford Research Ethics Committee A (reference 09/H0604/20 and N17/01/013), University of Oxford Tropical Research Ethics Committee (ref. OXTREC 01–18), Stellenbosch University Health Research Ethics Committee (HREC ref. N17/01/013), and the University of the Free State Research Ethics Committee (HSREC ref. 203/2016). All research was performed in accordance with relevant guidance and regulations, and participants provided written informed consent.

Samples were transported to the research laboratory within 2–3 h of collection, spun to separate serum, and frozen in aliquots at −80 °C. We recorded patient demographics, ALT, presence of HIV and elastography scores from routine clinical and electronic patient records. The serum sample collection date was designated ‘baseline’, and metadata including ALT, elastography scores and markers for calculation of non-invasive liver scores were collected as close to baseline as possible, with a limit of 12 months either side of this date.

We calculated laboratory fibrosis scores using the following formulae:

APRI = (AST (IU/L) / AST ULN) / Platelets (10^9^/L).
FIB-4 = Age (years) x AST (U/L) / Platelets (10^9^/L) x √ALT (U/L).
AST upper limit of normal (ULN) was defined as 40 U/L, as per 2015 WHO guidelines.^36^

### Quantification of standard serum parameters

Platforms used to measure clinical laboratory biomarkers (HBeAg, HBV VL, ALT, AST, Platelet count) in each cohort are listed in Suppl. Table 1. Assays were undertaken on validated clinical diagnostic platforms according to the manufacturers’ instructions.

### HBcrAg measurement

Quantitative levels of HBcrAg were determined using the Lumipulse G HBcrAg chemiluminescent immunoassay (CLEIA) (Fujirebio, Tokyo, Japan) at King’s College Hospital NHS Foundation Trust, London, UK, according to the manufacturer’s instructions. The assay has a linear measurement range of 3–7 log_10_ U/ml, using 3 log_10_ U/ml as the lower limit of detection (as recommended by the manufacturer). Samples generating HBcrAg measurements > 7 log_10_ U/ml were diluted (1:5, 1:100 or 1:1000 in Tris Buffered Saline) before repeat testing.

### Thresholds for biomarkers and liver elastography

We used thresholds for analysis based on existing precedents and 2024 WHO guidelines,^8^ including HBV VL > 2000 IU/ml, > 20,000 IU/ml and > 200,000 IU/ml, which have been established clinically to determine eligibility for antiviral treatment and/or prophylaxis,^36^ and ALT thresholds for upper limit of normal (ULN) of 19 IU/L in females and 30 IU/L in males. We defined the presence of liver fibrosis based on FIB-4 ≥1.45, APRI > 0.5 or elastography score > 7kPa, while cirrhosis was defined as FIB-4 ≥3.25, APRI > 1 or elastography score ≥12.5 kPa.^8^ We considered HBcrAg thresholds based on those previously determined as associated with relevant HBV VL thresholds,^17^ where HBcrAg 3.6 log_10_ U/ml, 4.5 log_10_ U/ml and 5.3 log_10_ U/ml were predictive of HBV VL > 2000 IU/ml, > 20,000 IU/ml and > 200,000 IU/ml, respectively.^27^ Previous data from the Gambia established the HBcrAg POCT to have a HBcrAg laboratory cut-off of approximately ≥4.3 (log_10_ U/ml).^28^ Whilst acknowledging we did not undertake POCT in this population, we applied this threshold as a binary cut-off to analyse the relationship with liver health markers in each cohort. We also calculated the sensitivity and specificity of our estimated positive HBcrAg POCT in predicting clinically significant thresholds that are applied to inform intervention.

### Data analysis and statistical methods

We used R version 4.2.0 and GraphPad Prism version 10.1.0. We did not pool data for SA and UK cohorts, but analysed each independently, recognising that the genetic, clinical, epidemiological, and environmental landscape of our two settings is not comparable, with multiple unmeasured influences that could contribute to liver disease and infection outcomes. Instead, we compared the findings between the two settings to review whether similar assumptions and uses of HBcrAg can be applied uniformly in different geographical populations. We focussed primarily on those not taking NA therapy to assess the use of HBcrAg in making treatment decisions. The plan for analysis of this data, including the questions we are addressing, is shown in Fig. 1.

(i) ***Descriptive statistics***.
  We produced descriptive statistics for each cohort, including median and interquartile ranges for patient demographics and HBV biomarkers. We generated comparative statistics within each cohort using Pearson’s Chi-squared test and the Wilcoxon rank-sum test.
  We also undertook an analysis of two distinct subpopulations within each cohort, based on a specific rationale about the potential utility of HBcrAg in each:
  > **HBeAg-negative**: In individuals who test HBeAg-positive, HBcrAg levels are likely to be dominated by HBeAg and independent measurement of HBcrAg as a biomarker may therefore not add any additional value. However, we hypothesised that HBcrAg may provide useful additional characterisation within the HBeAg-negative subpopulations.
  > **Those not taking NA therapy:** For analyses investigating correlations between HBV VL and liver fibrosis markers with HBcrAg, we included only people currently not on antiviral (NA) therapy. This removes some confounders, including varying treatment duration and HIV status, and provides the most useful interpretation for practical application of these findings in the real world.
(ii) ***Univariable analysis of relationship between HBcrAg and other parameters***.
  Within each cohort, we described the distribution of HBcrAg and explored its relationship with individual demographic factors (age, sex), routine laboratory biomarkers (HBeAg, HBV VL and ALT) and liver fibrosis/cirrhosis scores (elastography, FIB-4 and APRI). When assessing VL and liver fibrosis/cirrhosis scores, we included only the untreated population.
(iii) ***Sensitivity and specificity with which HBcrAg predicts clinically important HBV VL levels***.
  Among untreated individuals, we assessed the sensitivity and specificity of previously defined HBcrAg levels (3.6, 4.5 and 5.3 log_10_ U/ml)^17^ and the published laboratory threshold of the POCT (4.3 log_10_ U/ml)^28^ to predict clinically important VL thresholds (2000, 20,000 and 200,000 IU/ml respectively), with particular focus on HBcrAg as a risk-stratification tool in a clinical setting to determine eligibility for perinatal NA prophylaxis.
(iv) ***Focused multivariable analysis***.
  In untreated individuals, we accounted for age, sex, HBeAg status, treatment status and HBV VL in a multivariable model to investigate the extent to which HBcrAg could predict the presence of liver inflammation (based on ALT), fibrosis and cirrhosis (based on elastography, FIB-4 and APRI scores).^37^ For each measure of liver outcome, we independently fitted a binomial generalised linear model with those covariates as main effects along with HBcrAg, cohort and their interaction, and binary liver outcome as the dependent variable. The strength (log-odds ratio) and significance of the association between HBcrAg and liver outcome (stratified by cohort and compared between cohorts) were calculated using bias-reduced scores by the brgrlm R package. We used complete cases only - patterns of missingness therefore varying between the different measures of outcome.

## Results

### Clinical and laboratory phenotypes of HBV infection in cohorts in the UK and SA

We generated HBcrAg measurements from 353 samples (142 from the UK and 211 from SA); (Table 1). Sex and age were comparable between cohorts (p > 0.05). However, as anticipated, the cohorts differed in other important ways according to the setting: 68% of SA participants were black African, in contrast to more diverse ethnicities among participants recruited in the UK (p < 0.001). Compared to the UK, a higher proportion of the SA population was living with HIV coinfection, HBeAg-positive and had HBV VL > 200,000 IU/ml (Table 1; all comparisons p < 0.001), although median HBV VL did not significantly differ between cohorts (p=0.3). Reflecting these differences (particularly based on HBeAg-positivity and HIV coinfection), a higher proportion of the SA cohort was receiving antiviral treatment (129/197, 65%) compared to the UK (42/137, 35%) (p < 0.001). Based on ALT and APRI measurement, liver inflammation was not significantly different between the UK and SA cohorts (p=0.4 for both). However, FIB-4 and elastography scores were both significantly increased in the SA population compared to the UK (p=0.043 and p=0.025, respectively).

These findings, in addition to differences in environmental exposures, host and viral genetics, clinical, biological and treatment landscapes between settings, underpin our subsequent analysis approach, in which each cohort is assessed independently.

### Distribution of HBcrAg levels in two different settings

**Fig. 2:**
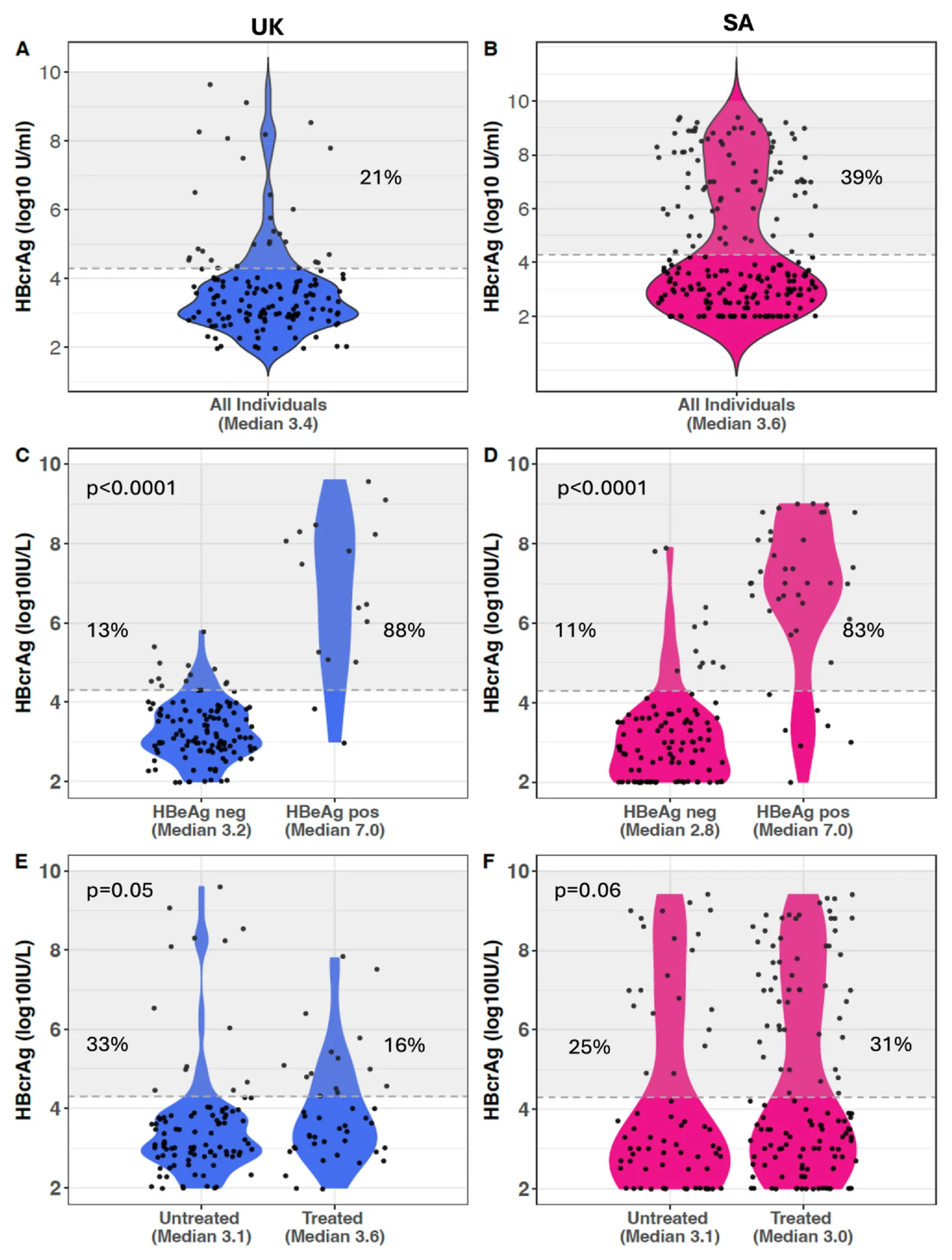
HBcrAg distribution in adults living with chronic hepatitis B (CHB) in South Africa (SA) and the United Kingdom (UK): A: UK cohort, all individuals, B: SA cohort, all individuals, C: UK cohort split by HBeAg status, D: SA cohort split by HBeAg status, E: UK cohort split by treatment status, F: SA cohort split by treatment status. p-values present Mann-Whitney-U test for significance using quantitative HBcrAg data. Grey areas represent the population who would be identified by a positive POCT (HBcrAg≥4.3 log U/ml); indicated next to each violin is the percentage of samples meeting this threshold. The x-axis labels include the median HBcrAg value for each column of data.

The distribution of HBcrAg varied between UK and SA populations (Table 1, Fig. 2A and B), and a greater proportion of those in the SA cohort had HBcrAg ≥5.3 log_10_ U/ml (70/211 (33.2%) vs 13/142 (9.1%) in UK, p < 0.001, Table 1). When estimating the HBcrAg POCT results (using the published threshold of 4.3 log_10_ U/ml), 30/143 (21.1%) of the UK population would test POCT positive compared to 82/211 (38.9%) of the SA population (p=0.0005, Table 1 and Fig. 2A and B). However, despite these differences, median HBcrAg was comparable between the UK and SA populations (3.40 log_10_ U/ml vs 3.57 log_10_ U/ml respectively, p=0.1, Table 1). On univariable analysis, HBcrAg was not associated with sex (p > 0.05), but had a significant negative association with age in both cohorts (p=0.01 in UK and p=0.004 in SA, Table 2), which is expected, in keeping with the biology of HBeAg loss over time.

As expected, in both cohorts, HBcrAg significantly differed between HBeAg-positive and -negative groups (p < 0.001), with 87.5% of HBeAg-positive samples meeting the POCT threshold in the UK cohort, and 82.5% in SA (Fig. 2C and D). In the HBeAg-negative population, 12.5% (UK) and 11.0% (SA) individuals would be predicted to test HBcrAg-positive on POCT. Median HBcrAg was significantly higher in those living with HIV co-infection compared to HIV-negative participants in SA (3.9 vs 3.3 U/ml, p < 0.001; Table 2). As HIV infection was infrequent in the UK cohort, we could not investigate any influence of HIV coinfection. HBcrAg was not associated with treatment status in either cohort (Fig. 2E and F).

### Risk stratification using HBcrAg based on correlation with HBV VL

**Fig. 3:**
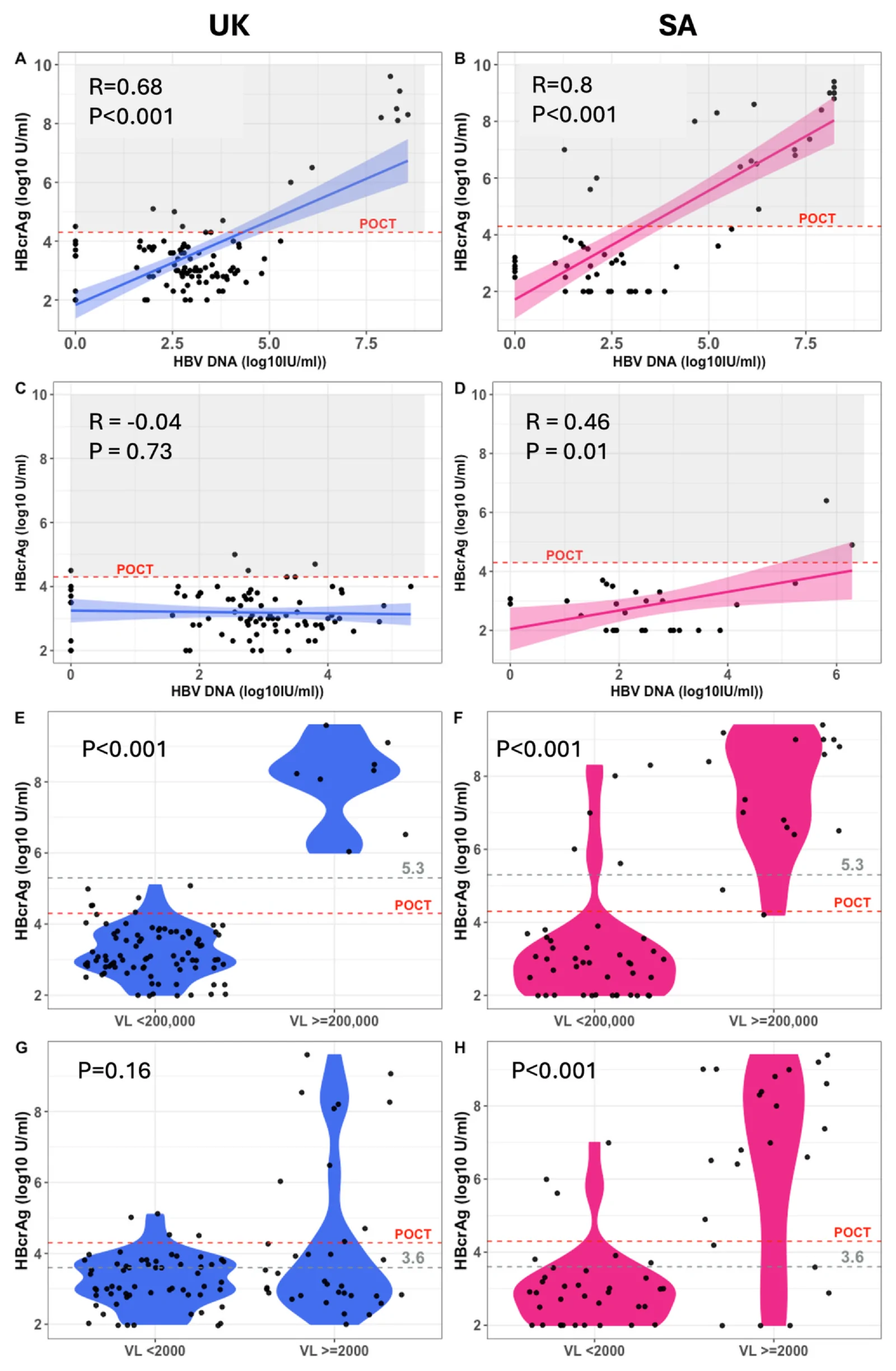
Relationship between HBcrAg and HBV viral load (VL) in untreated adults living with chronic hepatitis B (CHB) in South Africa (SA) and the United Kingdom (UK): A: UK all individuals, B: SA all individuals, C: UK HBeAg negative individuals only, D: SA HBeAg negative individuals only, E: UK cohort split by HBV VL < 200,000 IU/ml vs ≥200,000 IU/ml, F: SA cohort split by HBV VL < 200,000 IU/ml vs ≥200,000 IU/ml, G: UK cohort split by HBV VL < 2000 IU/ml vs ≥2000 IU/ml, H: SA cohort split by HBV VL < 2000 IU/ml vs > 2000 IU/ml. For panels A-D Pearson’s correlation is shown, panels E-H, Mann-Whitney-U test for significance is shown. Dotted red lines represent the HBcrAg POCT threshold (HBcrAg ≥4.3 log U/ml) with the shaded grey area in A-D showing all those points that would test positive; in panels E-H dotted grey lines represent the relevant HBcrAg threshold.

Considering only untreated individuals, there was a significant positive correlation between HBcrAg and VL in both the UK and SA cohorts (R=0.68 and 0.8, respectively, p < 0.001 for both; Fig. 3A and B). However, in the UK cohort, this correlation was lost when considering only the HBeAg-negative population (Fig. 3C and D). In both cohorts, there was no significant difference in median HBcrAg between untreated individuals with undetectable vs detectable VL (Suppl Figures 1A and 1B).

When considering the use of HBcrAg in informing treatment decisions, we evaluated its ability to identify those with VL > 2000 IU/ml and > 200,000 IU/ml (thresholds based on the 2024 WHO guidelines^8^). HBcrAg ≥5.3 log_10_ U/ml was 100% sensitive and specific at identifying those with VL > 200,000 IU/ml in the UK population but performed less well in the SA population (where it was 88% sensitive and specific) (Table 3). There was a statistically significant difference in median HBcrAg between those with VL ≥200,000 and < 200,000 in both cohorts, p < 0.001 (Table 3, Figs. 3E and 3F, Suppl Figure 2).

In both cohorts, the performance of lower HBcrAg levels in predicting defined lower HBV VL thresholds generally declined, particularly in the UK setting, with sensitivity for detecting VL 20,000 IU/ml of 73% in the UK and 89% in SA, and for VL 2000 IU/ml 43% in the UK and 83% in SA (Table 3). In SA there was still a significant difference in HBcrAg between those with HBV VL > 2000 IU/ml and < 2000 IU/ml **(**p < 0.001, Fig. 3H), however this was not the case in the UK (Fig. 3G).

The reported HBcrAg POCT threshold of 4.3 log_10_ U/ml could not accurately identify those with HBV VL > 2000 IU/ml in either the UK or SA (sensitivity 31% and 74% respectively, Table 3), although a greater proportion of those with VL > 2000 IU/ml had HBcrAg above this threshold in both populations (Fig. 3G and H). In contrast, HBV VL > 200,000 IU/ml (associated with the highest risk of vertical transmission) could be reliably identified by the POCT threshold in the UK population (100% sensitive and 92% specific), but less so in the SA population (94% sensitive and 86% specific) (Table 3, Fig. 3E and F, Suppl Figure 2).

### Association between HBcrAg and liver inflammation or fibrosis (off treatment)

**Fig. 4:**
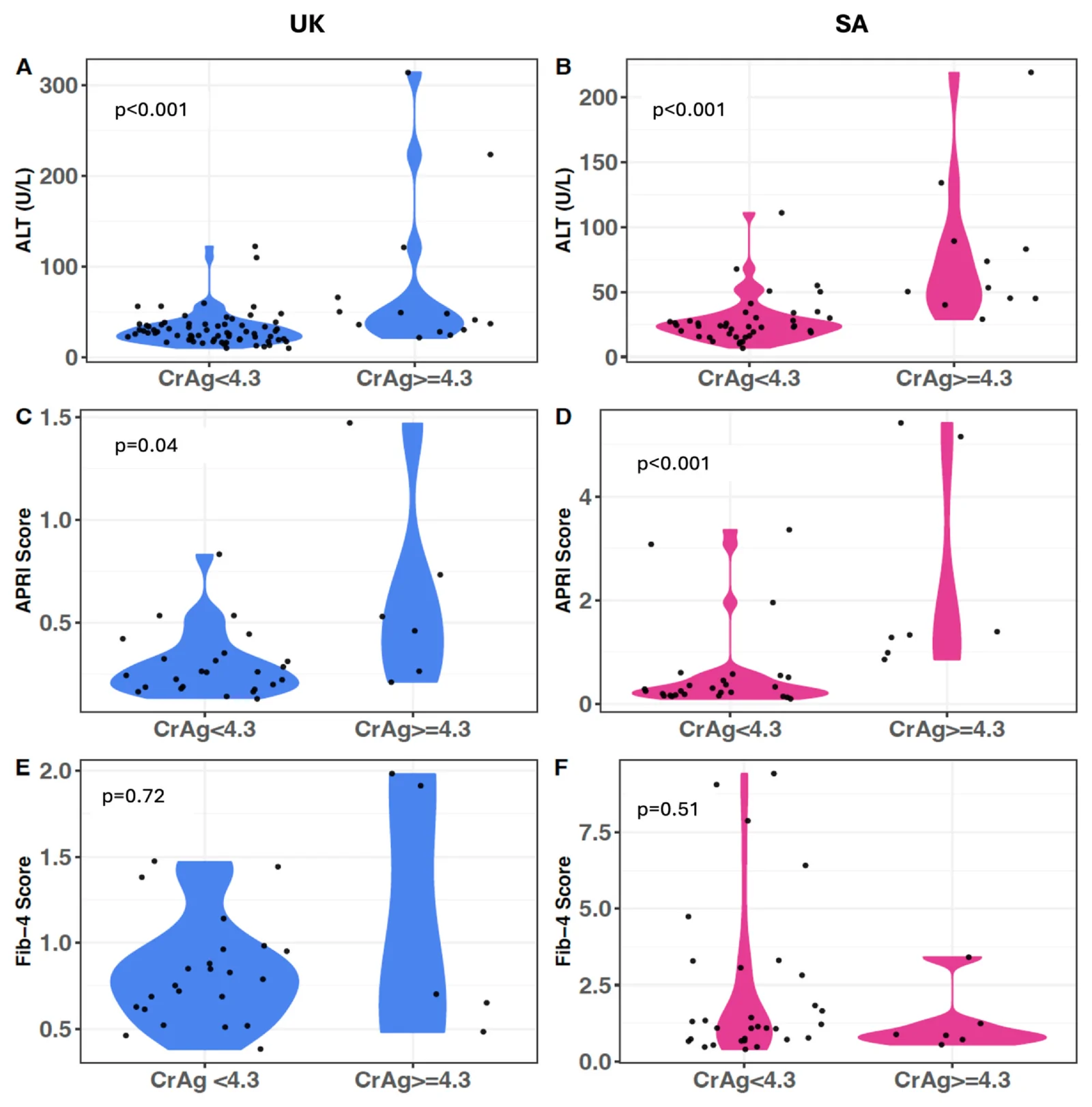
Relationship between HBcrAg POCT threshold of 4.3 log_10_U/ml and liver disease markers in adults with chronic, untreated HBV infection. UK data is shown in the left column (blue) and SA data in the right column (pink). A, B: HBcrAg result vs ALT; C, D: HBcrAg result vs. APRI; E, F: HBcrAg result vs. FIB-4 score.

In the untreated population, we observed a significant positive correlation between HBcrAg and both ALT and APRI scores in UK and SA populations (ALT: R=0.42 (UK), and 0.62 (SA), p < 0.001 for both, APRI: R=0.4, p=0.03 (UK) and R=0.45, p = 0.008 (SA), Table 2). However, there was no significant correlation between HBcrAg and liver stiffness scores assessed by elastography or FIB-4 in either cohort (Table 2). Median ALT and APRI scores were significantly higher in those with HBcrAg above this threshold in both populations (p < 0.05 for all, Fig. 4A–D), whereas FIB-4 and elastography scores did not significantly differ in either population (Fig. 4E and F).

Still in the untreated population, we applied a multivariable model analysis to determine the magnitude of association of HBcrAg with different liver health indicators across our study populations. This model accounted for covariates identified as potential confounders or predictors of outcome in univariable analysis, including age, sex, HBeAg status and HBV VL. This analysis showed the odds of different liver outcomes were neither significantly correlated with HBcrAg, nor were they different between the cohorts (Suppl Table 2).

## Discussion

We investigated the extent to which HBcrAg could improve clinical assessment, including considering its role if made accessible as a POCT, in two geographically distinct populations. We evaluated its relationship with demographic and clinical parameters, especially those that are relevant to treatment stratification.

A previous study on HBcrAg in Africa (PROLIFICA cohort in the Gambia) reports a laboratory median of 4.0 log_10_U/ml,^27^ which is comparable to both our UK and SA cohorts (3.4 and 3.57 log_10_U/ml, respectively). Only 13% of the Gambia population was HBeAg positive, compared to 11.3% in the UK cohort and 18.9% in our SA cohort. As expected, HBcrAg significantly positively correlated with HBeAg status and with HBV VL in both our study populations; correlation with VL persisted on excluding the HBeAg-positive population in SA, in line with previous observations from the Gambia.^27^ The higher HBcrAg found in those living with HIV was likely due to a higher proportion being HBeAg positive than those without HIV (33% vs 24%).

### Clinical utility of HBcrAg in risk assessment

Our observations illustrate the persistence of HBcrAg as a quantifiable biomarker even when HBV VL is low or undetectable. Given the overlap in proteins measured by testing for HBeAg and HBcrAg, the latter could be considered as a more sensitive assay that could supersede conventional HBeAg testing. Elastography score and ALT were previously correlated with HBcrAg levels in untreated individuals in the Gambian study^27^; correlation between HBcrAg and ALT was recapitulated in both our study settings, but this was not the case for elastography scores. Differences here could be explained by the low percentage of people with elevated elastography scores that suggest cirrhosis in our populations (5.7% and 7.8% for the UK and SA, respectively) compared to the previously investigated cohort in the Gambia (55%); our study is thus relatively under-powered. We found a positive correlation between HBcrAg and APRI; however, not for FIB-4 scores. When applying a multivariable model and accounting for confounding factors, these associations were lost.

While we did not directly evaluate POCT in this study, by applying the equivalent quantitative HBcrAg threshold of 4.3 log_10_ U/ml, we demonstrate that a positive POCT could potentially identify those with significant liver disease based on ALT and APRI. This was also the case in our Kenyan study directly evaluating the POCT, where those with a positive POCT had significantly higher ALT and APRI scores than those with a negative POCT.^29^ Here, the POCT threshold would be highly sensitive at identifying those eligible for perinatal antiviral prophylaxis in the UK population, as was also the case in Kenya, but less true in SA. Of note, the population in Kenya was more similar to that of the UK, with a low rate of HBeAg positivity and little HIV co-infection. These results demonstrate that HBcrAg POCT needs to be evaluated in distinct populations to build a use case, and different population thresholds may be needed.

### Resource impact of HBcrAg

Currently, HBcrAg CLEIA is not available in most settings and comes at a high cost, and running serial dilutions added to the cost of our laboratory assessment of samples with HBcrAg above the upper limit of quantification (although this refined quantification of high levels may not be necessary if the assay were to be deployed in more routine practice). The HBcrAg POCT is currently only available for research purposes, but given the existing literature and these new results, it may have promise for clinical use when other methods of stratification are unavailable. Costs of procuring the POCT must be balanced against cost and availability of other established monitoring tools that require laboratory facilities, and striking a balance between ongoing investment to develop and evaluate new tools, while avoiding unnecessary cost and complexity. The POCT can be stored at room temperature, does not require specialist equipment and can be done in the field. As turnaround times for diagnosis are often days (and may be weeks in some settings), POCT also adds the benefit of real-time evaluation (turnaround: 45 min), leading to prompt advice regarding treatment. A study that reported on screening of migrant populations in Belgium showed that the HBsAg POCT vs venepuncture testing led to a dramatic increase in linkage to care (from 34% to 86%).^30^ Hence, whilst we still need to refine the utility of HBcrAg, there is an emerging case that POCT can be associated with improved linkage to care.

### Limitations of this study

We measured HBcrAg in modestly sized cohorts, with significant missingness for some parameters (e.g. fibrosis scores only computed for < 50% of individuals which restricts our ability to apply imputation) and only on a cross-sectional basis. Small numbers in subgroups reduce statistical power for us to determine true effects, particularly in multivariable analysis. Most parameters were collected as close to baseline as possible, but we allowed a 12-month period on each side that could impact our analysis. We identified some important differences between our study populations in different geographical settings (demographics, HIV coinfection, treatment status), but there are also many unmeasured factors (differences in viral genotype, host genetics, comorbidity, treatment duration, environmental exposures, immune activation, microbiome) that are inter-related and may influence host and viral biomarkers and outcomes of infection. There are some discrepancies between our findings and existing literature.^15,38,39^ Differences between our two cohorts, and between this study and others, may be accounted for by (i) methodology used for fibrosis assessment (e.g. histological METAVIR analysis vs. circulating marker scores), (ii) location of study populations and (iii) disease stages, including HBeAg status.

We used the laboratory threshold of 4.3 log_10_U/ml as a substitute for a positive HBcrAg POCT, as its first evaluation in the Gambia suggested CLEIA and POCT HBcrAg sensitivity were comparable.^28^ However, we did not actually use POCT in this study and the previously set thresholds could differ in our geographical locations due to differences in HBeAg positivity or HBV genotypes. When trialled in the Gambia, there was no difference in the laboratory threshold between genotypes A and E; however, our UK cohort also includes people of Asian descent, in whom genotype B and C are more prevalent, which could explain the discrepancies.

Whilst large, longitudinal studies correlating HBcrAg with liver outcome have been done in other populations, this data is missing for Africa, where studies on HBcrAg are few, and primarily crosss-ectional, such as this one. Whilst these data are useful to understand the broad utility of HBcrAg and its value in different populations, data evaluating HBcrAg over time will primarily inform its utility in low-resource settings, for example, whether it can detect changes in liver fibrosis during NA therapy and how closely it can be used where other markers are not available.

## Conclusions

Based on this study, the distribution of HBcrAg levels differs between populations. In our cohorts, HBcrAg did not consistently predict liver inflammation or fibrosis; however, it was a strong predictor of HBV VL > 200,000 and may have a role in the stratification of interventions for prevention of vertical transmission, particularly if an affordable POCT becomes available. More data are needed to represent different population settings, and to determine the cost-benefit of introducing new testing at a time when global resource focus is on scale-up of diagnosis and treatment.

## Supplementary Material

Supplementary text

## Figures and Tables

**Table 1:** Characteristics of two cohorts of adults with chronic hepatitis B virus infection based in South Africa (SA) and the United Kingdom (UK).

Characteristic	Numbers (%) for whom data available	UK Cohort^a^ (%)	SA Cohort^a^ (%)	p-value^b^
**Total number**	353 (100)	142	211	
**Sex**	333 (94.3)			0.7
Female		56 (39.4)	92 (43.6)	
Male		66 (46.5)	119 (56.4)	
Unknown		20 (14.1)	0 (0.0)	
**Age (IQR)**	332 (94.1)	40 (36, 49) (N=123)	39 (34, 50) (N=209)	0.4
**Ethnicity**	314 (89.0)			**< 0.001**
Asian		52 (36.6)	2 (0.9)	
Black African		24 (16.9)	143 (67.8)	
Caucasian		29 (20.4)	4 (1.9)	
Mixed Race		0 (0.0)	59 (27.9)	
Other		0 (0.0)	1 (0.5)	
Unknown		37 (26.1)	2 (0.9)	
**HIV status**	339 (96.0)			**< 0.001**
Negative		126 (88.7)	92 (43.6)	
Positive		3 (2.1)	118 (55.9)	
Unknown		13 (9.2)	1 (0.5)	
**NA Therapy**	334 (94.6)			**< 0.001**
No		95 (66.9)	68 (32.2)	
Yes		42 (29.6)	129 (61.1)	
Unknown		5 (3.5)	14 (6.6)	
**Liver inflammation and fibrosis assessment**				
**ALT median** **(IQR), (U/L)**	285 (80.7)	29 (21, 40) (N=127)	26 (18, 44) (N=158)	0.4
**APRI score** **median (IQR)**	145 (41.1)	0.26 (0.20, 0.42) (N=45)	0.31 (0.19, 0.60) (N=100)	0.4
**FIB-4 score** **median (IQR)**	143 (40.5)	0.85 (0.64, 1.08) (N=42)	1.02 (0.67, 1.86) (N=101)	**0.043**
**Fibroscan** **median (IQR)**	153 (43.3)	5.2 (4.3, 6.5) (N=91)	5.7 (4.8, 7.8) (N=62)	**0.025**
**HBeAg status**	276 (78.2)			**< 0.001**
Negative		120 (84.5%)	100 (47.4%)	
Positive		16 (11.3%)	40 (18.9%)	
Unknown		6 (4.2%)	71 (33.6%)	
**HBV VL quantification (log_10_ IU/ml)**				
**HBV VL (log_10_ IU/ml) (IQR)**	312 (88.4)	2.54 (0.00, 3.37) (N=142)	2.15 (1.30, 4.91) (N=170)	0.3
**HBV VL Group, (log_10_ IU/ml)**	312 (88.4)			**< 0.001**
< 20		46 (32.3)	41 (19.4)	
20 - < 2000		58 (40.8)	73 (34.6)	
2000 - < 20,000		26 (18.3)	10 (4.7)	
20,000 - < 200,000		4 (2.8)	9 (4.3)	
≥200,000		8 (5.6)	37 (17.5)	
Unknown		0 (0)	41 (19.4)	
**HBcrAg values (log_10_U/ml)**				
**HBcrAg median (IQR)**	353 (100)	3.40 (2.90, 4.00)	3.57 (2.70, 6.80)	0.10
**HBcrAg Group**	353 (100)			**< 0.001**
< 3.6		85 (60)	111 (53)	
3.6 - 4.5		35 (25)	20 (9.5)	
4.5 - 5.3		9 (6.3)	10 (4.7)	
> 5.3		13 (9.2)	70 (33)	
**HBcrAg POCT** ^c^	353 (100)			**< 0.001**
Negative		112 (78.9)	129 (61.1)	
Positive		30 (21.1)	82 (38.9)	

a n (%); Median (IQR).

b Pearson’s Chi-squared test; Wilcoxon rank sum test; Fisher’s exact test.

c POCT – point of care test, estimated from a test that delivers a positive result at HBcrAg ≥4.3 log_10_U/ml.

**Table 2:** Correlation between HBcrAg and demographic, laboratory and imaging characteristics of adults living with chronic HBV infection in the United Kingdom (UK) and South Africa (SA).

	UK cohort (N=142)	SA cohort (N=211)
**All individuals** ***Demographic characteristics***		
Age	**R = - 0.22 (p = 0.014)**	**R = - 0.2 (p = 0.004)**
Sex	p = 0.67	p = 0.37
HIV Status	No data	**p < 0.001**
Treatment Status	p = 0.05	p = 0.06
**Untreated individuals only** ***Liver risk stratification/fibrosis assessment***		
HBeAg status	**p < 0.001**	**p < 0.001**
ALT	**R = 0.42 (p < 0.001)**	**R = 0.62 (p < 0.001)**
APRI	**R = 0.40 (p = 0.03)**	**R = 0.45 (p = 0.008)**
FIB-4	R = 0.22 (p = 0.27)	R = - 0.13 (p = 0.45)
Elastography score (kPa)	R = - 0.08 (p = 0.56)	R = 0.52 (p = 0.1)

Pearson’s correlation coefficient (R) is shown for continuous variables; Wilcoxon rank sum test is used to compare categorical variables.

**Table 3:** Sensitivity and specificity of HBcrAg levels as predictors of clinically significant HBV viral load (VL) levels in cohorts in the United Kingdom (UK) and South Africa (SA) in untreated individuals.

	HBV VL (lU/ml)	UK cohort		SA cohort	
***HBcrAg (log_10_U/ml)***		***Sensitivity***	***Specificity***	***Sensitivity***	***Specificity***
≥5.3	> 200,000	100%	100%	88%	88%
≥4.5	> 20,000	73%	94%	89%	90%
≥3.6	> 2000	43%	61%	83%	78%
***HBcrAg POC*** ***T*** ^a^		***Sensitivity***	***Specificity***	***Sensitivity***	***Specificity***
Positive	> 200,000	100%	92%	94%	86%
Positive	> 20,000	67%	92%	89%	90%
Positive	> 2000	31%	93%	74%	89%

a POCT – point of care test, based on a test that delivers a positive result at HBcrAg ≥4.3 log10U/ml.

## Data Availability

Metadata supporting the findings of this study are available at https://doi.org/10.6084/m9.figshare.27619773 (within figshare collection: https://doi.org/10.6084/m9.figshare.c.8002876).
