# Supplementary material for: Evaluation of Hepatitis B core-related antigen (HBcrAg) as a biomarker in cohorts from the United Kingdom and South Africa
Source: J Infect. Author manuscript; Available in PMC 2026 Sep 11. (PMC7619275; doi:10.1016/j.jinf.2025.106601)
Supplement: Supplementary file 1 [file EMS216195-supplement-Supplementary_text.docx]

**SUPPLEMENTARY MATERIAL**

**Evaluation of Hepatitis B core related antigen (HBcrAg) as a biomarker in cohorts from the United Kingdom and South Africa.**

**Downs et al.**

**CONTENTS**

**Supplementary table 1:** Summary of the laboratory platforms used for blood analysis during comparison of HBcrAg with other blood biomarkers in two cohorts, one from the United Kingdom (UK) and one from South Africa (SA).

**Supplementary table 2:** Odds ratios of liver disease outcomes with increasing HBcrAg in two cohorts living with chronic hepatitis B virus infection, one in the UK and one in SA. Odds ratios are determined within each cohort and then compared between cohorts.

**Supplementary figure 1:** HBcrAg in untreated individuals living with chronic hepatitis B virus infection in the UK and SA, with detectable vs undetectable HBV VL. The threshold for a positive HBcrAg point of care test is shown as a red dotted line.

**Supplementary Figure 2:** Relationship between HBcrAg and HBV VL in untreated individuals living with chronic hepatitis B virus infection in the UK and SA focussing on clinically important HBV VL thresholds.

**Suppl Table 1: Platforms used to measure routine clinical laboratory biomarkers in Oxford cohort (UK) and Stellenbosch/Bloemfontein cohort (SA)**

|  | **UK** | **SA** |
| --- | --- | --- |
| HBeAg | Abbott Architect i2000SR (Abbott laboratories, Chicago, IL; semi-quantitative^b^ | Cobas 6000 series e601 module analyzer (Roche Diagnostics GmbH, German |
| HBV DNA | Cobas Taqman assay (Roche Diagnostics, Manheim, Germany)^c^ | Cobas Ampliprep/Taqman tests (Roche Molecular Diagnostics, the Netherlands) |
| ALT | Abbott Architect c16000 or c8000 (Abbott laboratories, Chicago, IL) | Cobas 6000 series e601 module analyzer (Roche Diagnostics GmbH, Germany) |
| AST | Abbott Architect c16000 or c8000 (Abbott laboratories, Chicago, IL | Cobas 6000 series e601 module analyzer (Roche Diagnostics GmbH, Germany) |
| Platelet count | Sysmex XN20 and XN10 (Sysmex, USA) | Advia 2120i analyzer (Siemens Healthcare Diagnostics Inc, USA) |

^a^ HBsAg lower limit of detection = 0.05IU/ml,

^b^ HBeAg reports binary results (using 1 Paul Ehrlich Unit (PE) as the cut off for positive / negative.

^c^ Serum HBV DNA was also quantified for the Oxford dataset using quantitative PCR, Cobas Taqman assay (Roche Diagnostics, Manheim, Germany) to ensure values were accurate at the time of HBcrAg measurement in case of any DNA degradation over time. The quantification range for HBV VL was between 40 and 1.7 x 10^8^ IU/ml.

|  | **UK cohort** | **SA cohort** | **UK vs SA** |
| --- | --- | --- | --- |
| ***Elastography*** | | | |
| **Fibrosis** | -0.12 (p=0.84) | -0.06 (p=0.93) | 0.06 (p= 0.93) |
| **Cirrhosis** | -0.65 (p=0.33) | -0.28 (p= 0.69) | 0.37 (p= 0.56) |
| ***APRI*** | | | |
| **Fibrosis** | 1.04 (p=0.48) | 0.60 (p=0.66) | 0.44 (p= 0.58) |
| **Cirrhosis** | 0.19 (p=0.91) | 0.02 (p=0.99) | 0.17 (p= 0.82) |
| ***FIB-4*** | | | |
| **Fibrosis** | 0.53 (p=0.75) | 0.26 (p=0.85) | 0.27 (p= 0.73) |
| **Cirrhosis** | 0.25 (p=0.86) | -0.08 (p=0.95) | 0.33 (p=0.68) |
| ***ALT - Liver Inflammation*** | 0.48 (p=0.21) | 0.32 (p=0.39) | 0.16 (p=0.61) |

**Supplementary table 2:** Odds ratio of increasing HBcrAg with liver disease outcomes accounting for age, HBeAg and HBV VL in an untreated population living with chronic hepatitis B infection in the UK and SA. Odd ratios (natural logarithm scale) are displayed with associated p-values for each cohort or for the ratio of ratios in between the cohorts.

**Supplementary figure 1: HBcrAg in untreated individuals with detectable vs undetectable HBV VL.** Shown for two cohorts living with chronic hepatitis B virus infection, one from the UK and once from South Africa. A: Individuals with HBV VL >20 and ≤20 IU/ml in UK cohort; B: Individuals with HBV VL >20 and ≤20 IU/ml in SA cohort. Dotted red lines represent the HBcrAg POCT threshold (HBcrAg ≥4.3 log U/ml) with the shaded grey area showing all those points that would test positive. Mann-Whitney-U test for significance is shown.

**UK	SA**

**Supplementary figure 2: Relationship between HBcrAg and HBV VL in untreated individuals focussing on clinically important HBV VL thresholds.** Shown for two cohorts living with chronic hepatitis B virus infection, one from the UK and once from South Africa. A: In the UK population; B: In the SA population. Black vertical lines indicate HBV VL of either 2000 IU/ml or 200,000 IU/ml. Pearson’s correlation is shown between HBcrAg and HBV VL. Dotted red lines represent the HBcrAg POCT threshold (HBcrAg ≥4.3 log U/ml) with the shaded grey area showing all those points that would test positive.

**UK	SA**
